# Non-aspirin non-steroidal anti-inflammatory drugs in colorectal cancer: a review of clinical studies

**DOI:** 10.1038/s41416-022-01882-8

**Published:** 2022-06-28

**Authors:** Farzana Y. Zaman, Suzanne G. Orchard, Andrew Haydon, John R. Zalcberg

**Affiliations:** 1 Department of Medical Oncology, The Alfred Hospital, Alfred Health, Melbourne, VIC Australia; 2 School of Public Health and Preventive Medicine, Department of Epidemiology and Preventive Medicine, Monash University, Melbourne, VIC 3004 Australia; 3 Head of Cancer Research Program, School of Public Health and Preventive Medicine, Faculty of Medicine, Monash University, Melbourne, VIC Australia

**Keywords:** Colorectal cancer, Cancer prevention

## Abstract

Colorectal cancer (CRC) chemoprevention is an area of interest. Non-steroidal anti-inflammatory drugs (NSAIDs) are anti-inflammatory agents which have been identified as cancer chemoprevention agents given that inflammation is thought to contribute to tumorigenesis. Most studies have demonstrated that the NSAID, aspirin, plays a beneficial role in the prevention of CRC and colonic adenomas. Non-aspirin NSAIDs (NA-NSAIDs) have also been studied in CRC chemoprevention. There is increasing literature around their role in pre-cancerous polyp prevention and in decreasing CRC incidence and CRC-related outcomes in certain high-risk subgroups. However, the use of NA-NSAIDs may be accompanied by increased risks of toxicity. Further studies are required to establish the associations between concurrent aspirin and NA-NSAID use, and CRC-related outcomes.

## Introduction

In Australia, colorectal cancer (CRC) is estimated to be the fourth most commonly diagnosed cancer in 2020, and remains the second most common cause of cancer death [1]. CRC screening programmes, such as Australia’s National Bowel Cancer Screening Program, have contributed to decreased mortality [2]. Whilst secondary prevention strategies aimed at diagnosing CRC in its earliest stages, have led to improved outcomes via early detection, screening does not necessarily prevent the development of cancer. Over the last few decades, there has been an increasing interest in the primary prevention of cancers through modification of lifestyle factors (such as avoidance of smoking and heavy alcohol use, improved diet and increased physical activity) and chemoprevention—using pharmacological agents to prevent or delay cancer onset by blocking the initiation of carcinogenesis or suppressing tumour promotion and progression.

Non-steroidal anti-inflammatory drugs (NSAIDs) have been identified as chemoprevention agents. NSAIDs, including aspirin, are commonly used for inflammatory conditions and cardiovascular disease. Their mechanism of action is through inhibition of cyclo-oxygenase (COX) and prostaglandin E2 pathways, as well as via COX-independent pathways. Given the well-known anti-inflammatory effects of these drugs and the understanding that inflammation may lead to tumourigenesis, it is plausible that NSAIDs would play a beneficial role in cancer prevention and treatment. Biological mechanisms involved in the potential anticancer effects of NSAIDs include enhancing immune responses, inducing apoptosis and inhibiting angiogenesis [3].

The role of aspirin in CRC chemoprevention has been well-studied and widely reported. Systematic reviews and meta-analyses generally conclude that aspirin use is associated with a decreased incidence of colonic adenomas [4–6], and CRCs [7] in young- to middle-aged populations. Indeed, there is robust evidence for low- and high-dose aspirin (the latter targeting COX2 compared to targeting COX-1 with low-dose aspirin, in addition to COX-independent pathways) in the prevention of adenomas in hereditary conditions such as familial adenomatous polyposis (FAP) [8, 9] or Lynch syndrome [10]. Both conditions involve specific genetic mutations that pre-dispose individuals to CRC. There is also evidence supporting the association between aspirin use and reduced CRC-related mortality in certain subgroups, such as tumours overexpressing COX2 and proximal CRCs [11, 12]. However, conflicting findings were demonstrated in the recently published Aspirin in Reducing Events in the Elderly (ASPREE) Trial [13]. ASPREE demonstrated higher all-cause mortality among healthy older adults who received daily low-dose aspirin as primary prevention, compared to those who received a placebo [14]. The main contributor to this was a greater risk of death attributed to cancer, accompanied by a higher incidence of cancer in the aspirin group for late-stage cancers. In particular, at a median follow-up of 4.7 years, aspirin was associated with an elevated CRC mortality rate, though not CRC incidence [14, 15]. This is in contrast to previously published literature on the association between aspirin and decreased mortality related to CRC [11], and other cancers [16], although the bulk of this work has been conducted on young- to middle-aged or high-risk populations. It has been postulated that the concurrent use of non-aspirin NSAIDs (NA-NSAIDs) may need to be explored further to understand these results.

There is an increasing body of literature exploring connections between non-aspirin NSAIDs (NA-NSAIDs) and adenoma and CRC chemoprevention. This review aims to understand the potential roles of NA-NSAIDs, alone or in combination with aspirin, in mitigating the risk of polyposis, CRC incidence and cancer-related outcomes. It will also review the data surrounding the safety of NA-NSAIDs in this specific setting.

## NA-NSAIDs in the polyp prevention

Similar to the data pertaining to aspirin, much of the evidence for NA-NSAIDs in colorectal neoplasia chemoprevention comes from studies of patients with an elevated baseline risk of CRC. This includes specific subgroups such as individuals with previous adenomas or polyps, hereditary polyposis syndromes such as FAP and Lynch syndrome. Whilst only 5% of polyps will evolve into CRC, 95% of CRCs will arise from adenomatous polyps [17]. Notably, polyps are an early event in the carcinogenic pathway. Therefore, chemopreventive actions of NSAIDs in the prevention of polyp formation may differ to that in CRC. While most observational studies in this area have looked at the effect of all NSAIDs, there are numerous randomised control trials (RCTs) of NA-NSAIDs, such as the selective COX2 inhibitors (COX-2i) (Table 1).

**Table 1:** Non-aspirin NSAIDs in polyp and adenoma prevention.

Study	Design	Patient population	Drug dose and duration	Indication	Key results	Adverse events
Tangrea et al. [6]	Prospective cohort study	Individuals with previous adenomas (*n* = 1905)	Any NSAIDs including aspirin	Adenoma recurrence	OR 0.77 (95% CI: 0.63–0.95)	
Murff et al. [40]	Case control study	No previous adenoma, age 40–75 years	NA-NSAIDs and aspirin	Adenomatous polyp incidence	NA-NSAIDs associated with reduced risk OR 0.67 (95% CI: 0.53–0.86)	
Shaw et al. [18]	Cross-sectional study	Individuals with elevated^#^ and average risk of CRC (*n* = 2548)	Any NSAIDs	High-risk adenomatous polyps incidence	OR 0.65 (95% CI: 0.47–0.89)	
Steinbach et al. [19]	RCT	FAP (*n* = 77)	Celecoxib 100 mg BD or 400 mg BD or placebo for 6 months	Polyp burden and number	Reduction with celecoxib 400 mg BD Polyp burden: 30.7% reduction (celecoxib 400 mg BD) vs. 4.9% (placebo) Mean number of polyps: 28% reduction (celecoxib 400 mg BD) vs. 4.5% (placebo)	
Giardiello et al. [21]	RCT	FAP (*n* = 41, age 8–25 years)	Sulindac 75 mg or 150 mg BD or placebo for 48 months	Adenoma incidence	43% (sulindac) vs. 55% (placebo), *P* = 0.54 No significant difference in mean number of size of polyps.	Increased AEs G1-2 93% but no significant differences between groups
Baron et al. [24]	RCT	Previous adenoma (*n* = 2587)	Rofecoxib 25 mg daily or placebo for 3 years	Adenoma recurrence	41% (rofecoxib) vs. 55% (placebo), RR 0.76 (95% CI: 0.69–0.83)	Increased risk of upper gastrointestinal, thrombotic and cardiovascular adverse events with rofecoxib
Bertagnolli et al. [23]	RCT	Previous adenoma (*n* = 2035)	Celecoxib 200 mg BD or 400 mg BD or placebo for 3 years	Adenoma incidence	59% (celecoxib 200 mg) vs. 60.1% (celecoxib 400 mg) vs. 68.4% (placebo)	Increased risk of cardiovascular and thrombotic AEs (RR 1.6 for 200 mg, RR 1.9 for 400 mg)
Arber et al. [22]	RCT	Previous adenoma removal (*n* = 1561)	Celecoxib 400 mg daily or placebo for 3 years, then 2 years off	Cumulative adenoma recurrence at year 5	51.4% (celecoxib) vs. 57.5% (placebo), *P* < 0.001	Increased risk of renal AEs/hypertension (RR 1.35), general vascular AEs (RR 1.34) and cardiac AEs (RR 1.59)
Rostom et al. [4]	Systematic review of RCTs, case control and cohort studies	Adults at average or higher risk of CRC^*^	NA-NSAIDs (COX2 inhibitors) and aspirin	Adenoma incidence	NA-NSAIDs reduced adenoma incidence (cohort RR 0.64, case control RR 0.54) COX2 inhibitors reduced adenoma incidence (RCT RR 0.72)	
Dulai et al. [25]	Systematic review of 15 RCTs	Previous colorectal neoplasia	NA-NSAIDs and aspirin	Prevention of advanced metachronous neoplasia	NA-NSAIDs: OR 0.37 (95% CI: 0.24–0.53)	NA-NSAIDs associated with the highest risk of AEs (HR 1.23)

*NSAID* non-steroidal anti-inflammatory drug, *NA-NSAID* non-aspirin non-steroidal anti-inflammatory drug, *RCT* randomised-controlled trial.

^#^First-degree relative with CRC, history of polyps, positive faecal occult blood test or faecal immunohistochemical test.

*Personal or family history of adenoma, family history of sporadic CRC; excluding patients with FAP or HNPCC or personal history of CRC.

This table summarises the key studies exploring the role of non-aspirin NSAIDs in polyp and adenoma prevention.

Epidemiological studies point at an inverse association between NSAID use and adenoma-related outcomes. A prospective cohort study by Tangrea et al. in 2003 examined the association between any NSAID use and recurrence of colorectal adenomas, among individuals with previous adenomas entering the Polyp Prevention Trial. There was a significant reduction in adenoma recurrence in NSAID users, with the greatest effect being seen in advanced polyps [6]. Correspondingly, a cross-sectional study of patients with an elevated baseline risk (first-degree family history of CRC, history of polyps or positive faecal occult blood test) undergoing screening colonoscopy demonstrated that use of any NSAIDs was associated with a decreased risk of high-risk adenomatous polyps. This effect was greater with daily, compared to monthly, NSAID use, although the dose was not reported [18].

RCTs of the COX-2i celecoxib, in patients with FAP, have demonstrated the beneficial effects of celecoxib on polyp reduction and polyp extent, compared to placebo. In these studies, the effect was statistically significant for higher doses of celecoxib (400 mg twice daily for 6 months) [19, 20]. In contrast, another smaller RCT of sulindac in a younger patient group with FAP (patients aged 8–25 years) demonstrated no significant difference in the mean number or size of polyps with sulindac use; nor was there any impact of the rate of adenoma development [21].

The Prevention of Sporadic Adenomatous Polyps (PreSAP) RCT of celecoxib 400 mg daily for 3 years followed by 2 years off, in patients with previous adenoma removal also demonstrated a reduced risk of adenoma incidence at 5 years [22]. Similarly, the Adenoma Prevention with Celecoxib (APC) Trial showed that adenoma incidence was decreased with celecoxib use for 3 years, when compared to placebo. A greater effect was seen with higher doses of celecoxib (400 mg twice daily for 3 years) [23]. Rofecoxib has also been studied and has demonstrated a reduced risk of adenoma recurrence in this patient population [24]. These RCTs did not explore the impact of NA-NSAID use and CRC incidence. It must be noted that in all studies, the use of COX-2i was associated with an increased risk of serious cardiovascular adverse events [22–24].

A systematic review by the US Preventive Services Task Force established that COX-2i in patients at average or higher risk of CRC reduced adenoma incidence with RR 0.72 [4]. In their systematic review of 15 RCTs of chemoprevention in individuals with previous colorectal neoplasia, Dulai et al. found that NA-NSAIDs were the most effective agents for preventing advanced metachronous neoplasia, when compared with placebo (OR 0.37 [0.24–0.53]) [25]. In comparison, low-dose aspirin had a more modest beneficial effect (OR 0.71). However, as seen previously, NA-NSAIDs use was accompanied by a lower safety profile than both high-dose and low-dose aspirin [25].

## NA-NSAIDs and colorectal cancer incidence

Evidence for NA-NSAIDs in mitigating CRC incidence predominantly comes from epidemiological studies exploring the effect of all NSAIDs (Table 2). Case control studies analysing NSAID use and CRC incidence have demonstrated mixed results. A case control study investigating NSAID use (defined as >4 tablets per week for >1 month) in healthy adults (mean age 62 years), showed that NA-NSAID use was inversely associated with CRC incidence (OR 0.74 [0.60–0.90]) [26]. A population-based nested case control study of UK health registry participants (median age 72 years [64–79]) showed that any NSAID use was associated with a lower risk of CRC incidence (OR 0.94 [0.88–1.00], *P* = 0.048) and that prolonged COX-2i use (≥25 prescriptions in 13–48 months) in particular was associated with a greater risk reduction (OR 0.34 [0.14–0.85]) [27]. Another nested case control study in Taiwan demonstrated similar findings, with a lower incidence of CRC in patients having had at least two prescriptions of any NSAID in the 13–48 months prior to the index date [28]. A Danish population-based case control study also showed that long-term, high-intensity (average daily dose ≥0.3) NA-NSAID use in healthy adults was associated with a substantial reduction in CRC risk (OR 0.57 [0.44–0.74]) [29]. On the other hand, a Swedish population-based study of adults taking regular NSAIDs (cumulative exposure ≥6 months) found that only aspirin and non-selective NSAIDs (SIR 0.74 [0.71–0.77]), not COX-2i, were associated with a decreased risk of gastrointestinal cancers including CRC. These contrasting findings may be related to the smaller population exposed to COX-2i (*n* = 17,948) compared to aspirin (*n* = 783,870) and non-selective NSAIDs (*n* = 566,209) in this study, and an overall younger population exposed to COX-2i (14.1% COX-2i-exposed versus 54.9% aspirin-exposed in ≥70 years) [30].

**Table 2:** Non-aspirin NSAIDs and colorectal cancer incidence.

Study	Design	Patient population	Drug dose and duration	Indication	Key results
Din et al. [26]	Case control study	Healthy adults, mean age 62 years	NA-NSAIDs, any NSAIDs, low-dose aspirin >4 tablets per week for >1 month	CRC incidence	NA-NSAIDs inversely associated with CRC incidence OR 0.74 (95% CI: 0.60–0.90)
Vinogradova et al. [27]	Nested case control study	Healthy adults, median age of 72 years	NSAIDs including COX2 inhibitors	CRC incidence	Prolonged use (≥25 prescriptions in 13–48 months) of COX2 inhibitors associated with reduction OR 0.34
Friis et al. [29]	Population-based cohort study	Healthy adults	Long-term, high-intensity NA-NSAIDs (average daily dose ≥0.3)	CRC incidence	NA-NSAID use associated with a reduction in risk, especially with long-term, high-intensity use of COX2 inhibitors: OR 0.57 (95% CI: 0.44–0.74)
Mahipal et al. [32]	Prospective cohort study	Postmenopausal women	NA-NSAIDs and aspirin	CRC incidence	NA-NSAIDs associated with a lower incidence HR 0.63 (95% CI: 0.58–1.00)
Ait Ouakrim et al. [35]	Prospective cohort study	Germline MMR mutations carriers	All NSAIDs—ibuprofen	CRC incidence	Reduced risk with ibuprofen use of 1 month to 4.9 years compared to <1 month of use (HR 0.38); reduction only in proximal CRCs
Wang et al. [31]	Prospective cohort study	Adults aged >50 years	All NSAIDs ≥4 days per week for ≥4 years	CRC incidence	Use of any NSAIDs associated with lower risk across all subgroups
Rostom et al. [4]	Systematic review of RCTs, case control and cohort studies	Adults at average or higher risk of CRC^*^	NA-NSAIDs (COX2 inhibitors) and aspirin	CRC and adenoma incidence	NA-NSAIDs reduced adenoma incidence (cohort RR 0.64, case control RR 0.54) and CRC incidence (cohort RR 0.61, case control 0.70). COX2 inhibitors reduced adenoma incidence (RCT RR 0.72)
Tomic et al. [34]	Systematic review and meta-analysis	Healthy adults aged 40 years and older	Regular NA-NSAID use^^^	CRC incidence	NA-NSAIDs reduced incidence in specific subgroups: -women OR 0.81 (95% CI: 0.67–0.98) -Caucasian OR 0.69 (95% CI: 0.55–0.87) -higher doses OR 0.82 (95% CI: 0.69–0.99) -distal colon cancers OR 0.78 (95% CI: 0.69–0.88)

*CRC* colorectal cancer, *NSAID* non-steroidal anti-inflammatory drug, *NA-NSAID* non-aspirin non-steroidal anti-inflammatory drug, *RCT* randomised-controlled trial.

^*^Personal or family history of adenoma, family history of sporadic CRC; excluding patients with FAP or HNPCC or personal history of CRC.

^Defined as ≥1 tablet ≥2 times per week for ≥1 month, any use in the past 5 years for ≥3 days per week for ≥3 months, or use on ≥60 days per year.

This table summarises the key studies exploring the role of non-aspirin NSAIDs in colorectal cancer incidence.

The VITAL prospective cohort study in healthy adults aged 50 years and over, concluded that high use of any type of NSAIDs (≥4 days per week for ≥4 years) was associated with a lower risk of CRCs across all subgroups, and was largely unmodified by other patient characteristics such as sex, body mass index (BMI), physical activity, smoking, alcohol use and dietary factors [31]. In postmenopausal women, another prospective cohort study demonstrated that NA-NSAID use was associated with a lower incidence of CRC [32]. This was driven by a reduction in proximal CRC, but no statistically significant effect was seen with distal or rectal cancers.

In their meta-analysis of 12 case control and cohort studies, Wang et al. demonstrated that regular use of aspirin or NA-NSAIDs was associated with a statistically significant reduced risk of incident CRC within all subgroups (OR 0.75 [0.71–0.79]). However, the protective effect of aspirin was lessened in patients with a heavy smoking history or elevated BMI [33]. A systematic review analysing RCTs and epidemiological studies found that NA-NSAIDs were associated with reduced CRC incidence in cohort and case control studies [4]. A recent meta-analysis by Tomic et al. investigated the association between regular NA-NSAID use (defined as ≥1 tablet ≥2 times per week for ≥ 1 month, any use in the past 5 years for ≥3 days per week for ≥3 months, or use on ≥60 days per year) and incident CRC risk, in a population aged 40 years or older [34]. Analysis of 23 cohort and case control studies involving over one million subjects only demonstrated significant protective effects of NA-NSAIDs in specific subgroups: women, Caucasian patients, individuals taking higher doses of NA-NSAIDs, and those with distal colon cancers [34].

In another cohort study of patients with germline mismatch repair gene mutations, there was an association between ibuprofen use and reduced risk of CRC (HR 0.25 [0.10–0.62]) [35]. In a recent case control study of adults at average risk of CRC, Chen et al found that regular NSAID (including aspirin) use was associated with reduced CRC, regardless of genetic profiles [36]. An earlier case control study of CRC cases in 2015 found that the use of NA-NSAIDs was associated with a lower risk of CRC, which differed according to variations at single nucleotide polymorphisms in chromosomes 12 and 15 [37]. Indeed, there is a growing body of evidence that genetic and molecular variations may impact upon the potential effects of NSAIDs in chemoprevention. This may be similar to the finding that regular aspirin use is associated with a lower risk of B-raf proto-oncogene (BRAF)-wildtype (WT) CRC, but not BRAF-mutant CRC [38]. Amitay et al. also observed a variation in CRC risk reduction by molecular subtype with any NSAID use [39]. While regular NSAID use was associated with a 31% lower risk of CRC in all patients, analysis by molecular subtype showed that the risk reduction was greater in microsatellite stable compared to microsatellite instability (MSI)-high cancers; in BRAF-WT compared to BRAF-mutant and in Kirsten rat sarcoma viral oncogene homologue gene (KRAS)-WT tumours. In MSI-high cases, the use of NSAIDs was associated with over a 70% risk reduction in KRAS- or BRAF-WT tumours, but no reductions were found in KRAS- or BRAF-mutant cases [39].

## Combination NA-NSAIDs and aspirin in CRC incidence and outcomes

Limited data exist on the effect of the combined use of NA-NSAIDs and aspirin on cancer risk. Previous studies have estimated that up to 27% of regular aspirin users simultaneously take NA-NSAIDs [40]. Based on studies pointing to the benefits of aspirin and NA-NSAIDs alone, coupled with our understanding of the potential biological mechanisms responsible for the chemopreventive effects of NSAIDs [41], it may be that combination therapy could have synergistic effects in particular subgroups.

Shebl et al. explored the independent and combined effects of aspirin and NA-NSAID use (defined as any frequency of use in the year prior) in inflammation-related cancers, in their prospective cohort study involving over 300,000 participants [42]. They demonstrated that the use of NSAID and NA-NSAIDs was associated with a lower risk of all inflammation-related cancers, infection-related cancers and obesity-related cancers, compared to aspirin alone and NA-NSAID alone. Models for specific cancer sites also showed that the use of aspirin and NA-NSAID in combination was associated with a reduced risk of cancers, including CRC [42].

Contrastingly, in further analysis of the APC trial, Bertagnolli et al. found that in the celecoxib 400 mg twice daily group, concurrent low-dose (≤325 mg alternative days or 162.5 mg daily) aspirin users had similar 5-year adenoma detection rates compared to participants using celecoxib alone (59.7% in aspirin users versus 60.4% in aspirin non-users). However, in the placebo group, concurrent aspirin use was associated with a statistically significant decrease in adenoma detection rates (66.9% in aspirin users versus 69.2% in aspirin non-users), suggesting that this beneficial effect is predominantly driven by NA-NSAID use or aspirin use, not both—in the presence of celecoxib, the benefit from aspirin appears to be mitigated. Murff et al. investigated the role of combination aspirin (either 81 mg or 325 mg) and NSAIDs for ≥3 days a week over 1 year, in patients with adenomatous polyps [40]. Similarly, they observed a reduced risk of colon adenomas in individuals regularly using aspirin or NA-NSAIDs, but no statistically significant difference in patients reporting both regular aspirin and NA-NSAID use [40].

A recent meta-analysis of eight RCTs and comparative studies exploring the risk of colorectal neoplasms and cardiovascular events after combined use of COX-2i and low-dose aspirin for 3 years found that COX-2i were associated with a 21% reduced risk of neoplasms in the first to third years of use. However, interestingly, they were counterproductive in the fifth year—compared to aspirin alone, COX-2i combined with aspirin for 3 years increased the risk of CRC by 80% [43]. These findings are summarised in Table 3.

**Table 3:** Non-aspirin NSAIDs and aspirin in combination in adenoma and colorectal cancer incidence and outcomes.

Study	Design	Patient population	Drug dose and duration	Indication	Key results
Murff et al. [40]	Case control	Healthy adults aged 40–75 years	Aspirin (81 mg or 325 mg daily) + NA-NSAIDs for at least 3 days a week over 1 year	Hyperplastic polyp risk	No reduction in risk with combination OR 0.65, 95% CI 0.34–124, *P* = 0.56
Shebl et al. [42]	Prospective study	Healthy adults	Aspirin + NA-NSAIDs	CRC incidence	Combination associated with reduced risk of CRC HR 0.73, 95% CI 0.66–0.79
Bertagnolli et al. [23]	RCT	High risk or previous adenoma	Celecoxib vs placebo for 3 years + concurrent low-dose aspirin (≤325 mg every other day or 162.5 mg daily	Adenoma recurrence	Reduction in adenoma incidence with concurrent aspirin: 60.4% (celecoxib 400 mg without aspirin) vs. 59.7% (with concurrent aspirin)
He et al. [43]	Meta-analysis	Healthy adults	COX2 inhibitors + low-dose aspirin for 3 years	CRC incidence	Compared to aspirin alone, COX2 inhibitors + aspirin for 3 years increased the risk of CRC by 80%

*NSAID* non-steroidal anti-inflammatory drug, *NA-NSAID* non-aspirin non-steroidal anti-inflammatory drug, *RCT* randomised-controlled trial.

This table summarises the key studies exploring the role of non-aspirin NSAIDs and aspirin in combination, in adenoma and colorectal cancer incidence and outcomes.

## NA-NSAIDs and CRC-related outcomes

Several studies have investigated the role of NA-NSAIDs in CRC-related outcomes (Table 4). Lipworth et al. undertook a population-based cohort study of mortality in ibuprofen users, which demonstrated a standardised mortality rate for CRC of less than 1.0, compared to the expected CRC-related mortality in the general population, three or more years after ibuprofen prescriptions [44]. This was associated with a slight increase in overall mortality in the first year of ibuprofen use—this appeared to be driven mostly by elevated all-cancer mortality, and gastrointestinal bleeding. It was proposed that this was confounded by patients with cancer utilising ibuprofen for analgesia. Mortality beyond the first year was largely driven by hypertension and diabetes.

**Table 4:** Non-aspirin NSAIDs and colorectal cancer-related outcomes.

Study	Design	Patient population	Drug dose and duration	Indication	Key results	Adverse events
Lipworth et al. [44]	Population based	Healthy adults	Ibuprofen for at least 1 year	CRC mortality	CRC SMR < 1.0 three or more years after ibuprofen	Increased mortality in 1st year of ibuprofen use due to gastrointestinal bleeding.
Din et al. [26]	Case control study	Healthy adults	NA-NSAIDs, any NSAIDs, low-dose aspirin	All-cause mortality or CRC survival	No effect of any NSAIDs	
Figueiredo et al. [47]	Prospective cohort study	Individuals diagnosed with CRC	Any NSAIDs	CRC-specific mortality	No effect of pre- or post-diagnosis NA-NSAID use	
Ng et al. [49]	Prospective cohort study	Resected Stage 3 CRC on adjuvant chemotherapy	Any NSAIDs including COX2 inhibitors	DFS, OS	COX2 inhibitors improved DFS: HR 0.47 (95% CI: 0.24–0.91) and OS: HR 0.26 (95% CI: 0.08–0.81) censored at 5 years	
Schak et al. [54]	Prospective cohort study	Individuals undergoing CRC surgery	Ibuprofen or diclofenac ≥2 days over 3 years	DFS, 5-year mortality	No effect	
Meyerhardt et al. [50]	RCT	Resected stage 3 CRC on adjuvant chemotherapy for 3 or 6 months	Celecoxib 400 mg daily or placebo for 3 years	DFS, OS	3-year DFS: 76.3% (celecoxib) vs. 73.4% (placebo), HR 0.89 (95% CI: 0.76–1.03, *P* = 0.12)	Increased risk of AEs with celecoxib compared to placebo: -Hypertension (14.6% vs. 10.9%) -G2 or higher increase in creatinine level (1.7% vs. 0.5%)
					5-year OS: 84.3% (celecoxib) vs. 81.6% (placebo), HR 0.86 (95% CI: 0.72–1.04, *P* = 0.13)	

*DFS* disease-free survival, *NSAID* non-steroidal anti-inflammatory drug, *NA-NSAID* non-aspirin non-steroidal anti-inflammatory drug, *OS* overall survival, *RCT* randomised-controlled trial.

This table summarises the key studies exploring the role of non-aspirin NSAIDs in colorectal cancer-related outcomes.

In another study of patients aged 20–74 years diagnosed with CRC, the use of any NSAIDs (≥2 times per week for ≥1 month) prior to diagnosis was associated with a 20% lower rate of CRC-related mortality [45]; although this effect was only seen in proximal CRCs and not in patients with rectal or distal CRCs. However, NA-NSAID use was not as important a factor as aspirin use for CRC survival. Further analyses suggested that the dose and duration of NSAID were more important in chemoprevention than the type of NSAID [45]. Likewise, in patients with germline mismatch repair mutations, the association between ibuprofen use and CRC outcomes was stronger with a longer duration of ibuprofen use (HR 0.38 for 1 month–4.9 years, versus HR 0.25 for over 5 years, compared to less than 1 month of use), suggesting that the beneficial effects of NA-NSAIDs are dependent on the duration of their use, at least in those with mismatch repair mutations, which are relatively common in sporadic cancers, and those with hereditary syndromes [35].

A cohort study of patients undergoing CRC surgery found that perioperative use of NA-NSAIDs (ibuprofen or diclofenac ≥2 days over 3 years) was associated with a reduced risk of CRC recurrence (HR 0.84). However, there was no effect on 5-year mortality or disease-free survival (DFS) [46]. Similarly, Figueiredo et al. recently showed that in patients already diagnosed with CRC, neither pre-diagnosis nor post-diagnosis use of NA-NSAIDs was associated with any improvements in CRC-specific mortality [47]. No effect of any NSAIDs, including NA-NSAIDs, on all-cause mortality or CRC-specific survival was seen in a case control study by Din et al. [26].

In a retrospective cohort study of patients with Stage 1–3 CRC aged ≥40 years, the use of any NSAID ( ≥ 1 prescription during the study period) was associated with a threefold decreased risk of recurrence and a sevenfold decreased risk of death. However, NA-NSAIDs were not studied separately [48]. In patients with resected Stage 3 CRC having adjuvant chemotherapy, a prospective observational study demonstrated that COX-2i were associated with improved DFS and overall survival (OS) at 5 years [49]. Based on this, Meyerhardt et al. undertook an RCT of celecoxib 400 mg daily for 3 years versus placebo in patients with Stage 3 CRC undergoing adjuvant chemotherapy for 3 or 6 months. In this interventional trial, celecoxib did not significantly improve DFS or OS, but came with an increased risk of hypertension and creatinine elevation [50]. It has been postulated that COX2-independent pathways may be more important for anti-neoplastic effects, and are better targeted by aspirin than selective COX-2i.

Indeed, the majority of studies involving NA-NSAIDs identified an elevated incidence of cardiovascular toxicity among patients with a high baseline cardiovascular risk who received NA-NSAIDs, and an increased risk of gastrointestinal bleeding—in keeping with the well-known side effects of NA-NSAIDs.

## NA-NSAIDs and toxicity

There is robust evidence supporting the use of NA-NSAIDs in high-risk populations for polyp prevention. The role of NA-NSAIDs as CRC chemoprevention in average-risk populations, and in mitigating CRC-related outcomes, is more equivocal. Many of these studies, including interventional trials, showed that NA-NSAID use was accompanied by an increased rate of adverse events.

The safety analysis of the APC trial found an increased risk of cardiovascular (RR 1.6) and thrombotic events (RR 1.9) for both low- and high-dose celecoxib [23]. This was particularly the case in patients with pre-existing atheroscelortic heart disease, which is a common comorbidity. Similarly, in the PreSAP trial, the beneficial effects of celecoxib appeared to be limited by a significantly increased risk of renal and hypertensive (RR 1.35 [1.09–1.68]), general vascular (RR 1.34 [1.08–1.68]) and cardiac (RR 1.59 [1.12–2.26]) adverse events. In fact, the safety signal noted in the APC trial led to the premature cessation of the PreSAP trial. Systematic reviews have concluded that NA-NSAIDs, and in particular COX-2i, increase the risk of serious cardiovascular events (RR 1.86 [1.33–2.59]) compared to placebo), and that NA-NSAIDs have the highest risk of adverse events, compared to low-dose aspirin (which is identified as being the safest) [25]. Rofecoxib in chemoprevention of colorectal adenomas, and in adjuvant CRC treatment trials was also associated with an increased risk of cardiovascular events [51, 52]. Gastrointestinal bleeding risk was also increased in the rofecoxib group compared to placebo. In their population-based study of mortality amongst ibuprofen users in Denmark, Lipworth et al. found that the increased mortality in the first year of ibuprofen use was due to gastrointestinal bleeding. Increased cancer mortality was also noted in the first year, and was attributed to patients with cancer using ibuprofen for analgesia [44]. It is unclear whether any of the cancer-related mortality was driven by increased gastrointestinal bleeding in gastrointestinal cancers.

In contrast, in the RCT of sulindac for primary chemoprevention of FAP, no significant differences in rates of adverse events were found between treatment and placebo groups [21]. However, this was a small trial involving a significantly younger population aged 8–25 years, compared to other trials, which may explain this anomaly. In a meta-analysis, He et al. found that combined COX-2i and aspirin use decreased the risk of cardiovascular and thromboembolic events, renal events and hypertension, compared to COX-2i alone [43]. It may be that the well-known cardiovascular health benefits of aspirin influence this finding.

Nonetheless, the clear increased cardiovascular and gastrointestinal bleeding toxicities associated with particular NA-NSAIDs may abrogate any additional benefits of these drugs in CRC chemoprevention. This is particularly pertinent as most trials demonstrating a beneficial effect of NA-NSAIDs in chemoprevention found that this effect was associated with the duration and dose of NA-NSAIDs. Notably, the most recent American Gastroenterological Association Clinical Practice Update on Chemoprevention for Colorectal Neoplasia advises against the use of NA-NSAIDs to prevent colorectal neoplasia in individuals at average risk for CRC, because of a substantial risk of cardiovascular events [53].

## Conclusion

There are many studies exploring the association between NA-NSAIDs and CRC in the literature. These range from epidemiological studies to randomised-controlled interventional trials. The role of NA-NSAIDs has been explored across the spectrum of CRC-related outcomes and adenoma prevention, from prevention of adenomas in high-risk patient populations, to their role in influencing CRC-related mortality. The majority of these studies support the role of NA-NSAIDs in polyp prevention in high-risk individuals. Large population-based studies suggest that there may be particular subgroups of healthy patients who benefit from NA-NSAIDs for chemoprevention—such as people aged ≥40 years, women, Caucasian patients, individuals taking higher doses of NA-NSAIDs and those with distal CRCs. There is also emerging evidence from observational studies that genetic and molecular variations may influence the chemoprevention effects of NA-NSAIDs in CRC. However, there are conflicting findings in the domain of CRC incidence and CRC-related outcomes. Nonetheless, the use of NSAIDs, particularly NA-NSAIDs, are associated with serious adverse events which may limit their use in a chemoprevention setting. The role of NA-NSAIDs in longer-term cancer-related outcomes such as survival has not been clearly established. Finally, further exploration is also required to elucidate the associations between concurrent aspirin and NA-NSAID use and cancer-related outcomes.

## Data Availability

Data sharing is not applicable to this article, as no datasets were generated or analysed during this study.
